# Polymicrobial sepsis and non-specific immunization induce adaptive immunosuppression to a similar degree

**DOI:** 10.1371/journal.pone.0192197

**Published:** 2018-02-07

**Authors:** Katrin Schmoeckel, Daniel M. Mrochen, Jochen Hühn, Christian Pötschke, Barbara M. Bröker

**Affiliations:** 1 Department of Immunology, Institute of Immunology and Transfusion Medicine, University Medicine, Greifswald, Germany; 2 Experimental Immunology, Helmholtz Centre for Infection Research, Braunschweig, Germany; Imperial College London, UNITED KINGDOM

## Abstract

Sepsis is frequently complicated by a state of profound immunosuppression, in its extreme form known as immunoparalysis. We have studied the role of the adaptive immune system in the murine acute peritonitis model. To read out adaptive immunosuppression, we primed post-septic and control animals by immunization with the model antigen TNP-ovalbumin in alum, and measured the specific antibody-responses via ELISA and ELISpot assay as well as T-cell responses in a proliferation assay after restimulation. Specific antibody titers, antibody affinity and plasma cell counts in the bone marrow were reduced in post-septic animals. The antigen-induced splenic proliferation was also impaired. The adaptive immunosuppression was positively correlated with an overwhelming general antibody response to the septic insult. Remarkably, antigen “overload” by non-specific immunization induced a similar degree of adaptive immunosuppression in the absence of sepsis. In both settings, depletion of regulatory T cells before priming reversed some parameters of the immunosuppression. In conclusion, our data show that adaptive immunosuppression occurs independent of profound systemic inflammation and life-threatening illness.

## Introduction

Sepsis is difficult to treat and causes high morbidity and mortality [1]. The dissemination of bacteria and their products elicits a systemic inflammation response (SIRS, systemic inflammatory response syndrome), resulting in multiple organ failure and shock [2,3]. This can be therapeutically addressed by adequate antibiotic therapy as well as supportive measures, such as hemodynamic stabilization. An even greater therapeutic problem is anti-inflammatory counter-regulation (CARS, compensatory anti-inflammatory response syndrome), which sets in after or even simultaneously with SIRS. During CARS, the anti-bacterial effector mechanisms are seriously impaired such that the immune system cannot clear the primary infection and is highly susceptible to secondary infections [2–4]. More than 70% of sepsis patients die because of immunosuppression, which in its extreme form is referred to as immunoparalysis [2,5–7].

It is well documented that in sepsis, many lymphocytes undergo apoptosis, which contributes to the hypoinflammatory milieu of CARS [8,9]. The innate response is also impaired, as reflected by reduced expression of HLA-DR on human monocytes [3,5,10] and diminished production of pro-inflammatory cytokines (IL-1α, IL-1β, IL-6, IL-12, TNF-α) *in vivo* and *in vitro*[11–16]. Additionally, the T cell response is dominated by Th2 and regulatory T cells [2,17].

The examination of immunosuppression in sepsis models requires that mice overcome the hyperinflammatory phase and that their immune response can be quantified over the course of the disease. Several groups have used cecal ligation and puncture (CLP) models with low (<10%–20%) or high (30–50%) lethality and combined them with a bacterial infection as a second hit [12,13,15,16,18]. Antibiotics (ertapenem) can improve survival of CLP [15]. The milder forms of CLP resulted in an early but transient impairment of the defense against the secondary infection [11–14,16,18], while highly lethal CLP reduced the immune response in the surviving animals for up to 30 days after induction of sepsis [15]. This was accompanied by a blunted innate immune response, with diminished production of pro-inflammatory cytokines (IL-1α, IL-1β, IL-6, IL-12, TNF-α) *in vivo* and *in vitro*[11–16].

The contribution of the adaptive immune system to immunosuppression remains a matter of debate [3,12,14,15,19–21]. Mohr *et al*. immunized animals between 1 and 14 days after mild CLP with ovalbumin (OVA) in alum and observed a reduction of the antigen-specific IgG response two days after CLP [20]. The underlying mechanisms remained elusive, however, because transfer of naïve, non-septic CD4^+^ T cells, B cells or dendritic cells did not restore immune competence [20].

Regulatory T cells are critical for immune homeostasis, because they limit inflammatory immune responses and the concomitant tissue damage. They have an essential role in the establishment and maintenance of peripheral immune tolerance. The discovery of a lineage-specific transcription factor, the forkhead box protein 3 (Foxp3) in 2003 was a milestone in immunology because it greatly facilitated identification of this T cell subset and enabled in depth analysis of its effector mechanisms. The majority of CD4^+^ Foxp3^+^ regulatory T cells (Tregs) originate in the thymus (thymus-derived Tregs, tTregs), the remainder are generated in the periphery (pTregs) by induced differentiation of Foxp3^-^ T cells. These two CD4^+^ Foxp3^+^ Treg subsets are also known as “natural” (nTregs) and “induced” (iTregs), respectively [22,23]. Upon interaction with antigen-specific B cells, CD4^+^ Foxp3^+^ Tregs can develop into follicular regulatory T cells (T_fr_), which control the humoral immune response developing in the germinal centers of peripheral lymphoid organs [24]. Foxp3 deficiency causes fatal autoimmune diseases in humans and mice. Effector mechanisms of Foxp3^+^ Tregs comprise cell contacts and anti-inflammatory cytokines, IL-10 and TGF-β [22]. Besides CD4^+^ Foxp3^+^ Tregs, there are Foxp3^-^ T cell subsets with anti-inflammatory properties. T helper 3 cells (Th3) and T regulatory 1 cells (Tr1) have prominent roles in maintaining mucosal tolerance. They exert their regulatory function primarily by the secretion of the anti-inflammatory cytokines TGF-β and IL-10, respectively [25]. Foxp3^+^ Tregs, T_fr_, Th3 and Tr1 all belong to the CD4^+^ T cell subpopulation. However, CD8^+^ T cells with regulatory potential also exist. They are Foxp3^-^, develop in the thymus and exert their anti-inflammatory effect by releasing both IL-10 and TGF-β [26].

This manuscript primarily addresses CD4^+^ Foxp3^+^ regulatory T cells (short: Tregs), which are by far the most extensively studied subset of T cells with regulatory function.

In sepsis, the fraction of Tregs is increased and the cells are activated [4,19,21], which may contribute to CARS. During early hyperinflammation, however, Tregs are required for the return to immune homeostasis. Animals depleted of Tregs at this stage were unable to resolve the inflammation and died from extensive tissue damage and multiple organ failure [27]. We propose that, conversely, in the later phase of sepsis, or even post sepsis, the activation and relative expansion of Tregs suppresses effective anti-infective immune responses. Therefore, this study analyzed the role of Tregs at the post-septic stage, where animals had overcome the bacterial insult and were again free of overt disease symptoms.

Besides the cellular adaptive immune response, an overwhelming humoral adaptive immune reaction is observed in sepsis [20,28]. We hypothesize that this phenomenon may interfere with the ability of the adaptive immune system to respond to novel stimuli (priming) because of cellular competition for limited niches. To test this, we have separated the exuberant antibody production from the severe disease caused by septic hyper-inflammation by exposing mice to numerous innocuous antigens simultaneously (non-specific immunization, [29]). We showed that this also resulted in a pronounced humoral response and interfered with subsequent antigen-specific priming in these non-septic, symptom-free animals.

Our results suggest that profound adaptive immune suppression can occur independent of the strong inflammation and severe disease symptoms that accompany polymicrobial sepsis.

## Materials and methods

### Ethics statement

All animal experiments were performed according to the German Animal Welfare Act (Deutsches Tierschutzgesetz) and the Federation of Laboratory Animal Science Associations (FELASA). The animal research protocol was approved by a committee of the responsible local animal protection authority (State Office for Agriculture, Food Safety and Fisheries Western Pomerania; numbers 7221.3–1.1-028/11, 7221.3–2.1-003/12, 7221.3–2.1-001/13). All surgery was performed under Ketamin/Xylazin anaesthesia, and all efforts were made to minimize suffering.

### Mice

C57BL/6 and DEREG female mice (depletion of regulatory T cells) from our own breeding were used at the age of 10 to 12 weeks (19–22 g). DEREG mice (C57BL/6 genetic background), which are heterozygous for the diphtheria toxin receptor (DTR)-eGFP transgene under the control of the *Foxp3* promotor, enable selective depletion of Foxp3^+^ Tregs [30]. C57BL/6 wild-type mice (WT) treated with diphtheria toxin (DT) or DEREG mice treated with PBS served as controls. The mice were housed in a conventional animal facility with sterile food and water *ad libitum* and were maintained by the Central service and Research Institute for experimental animals of the University Medicine Greifswald according to the guidelines of the German Animal Welfare Act.

### Peritoneal sepsis model—Acute peritonitis (AP)

The acute peritonitis model (AP) was used as sepsis model and carried out as described by Barrera *et al*. [31]. In brief, mice were anaesthetised with Ketamin/Xylazin (100 mg/10 mg per kg body weight), and an 18-gauge stent was inserted into the cecum opposite of the ileocecal valve and fixed to the gut. To compensate for fluid loss, animals received 500 μL of physiological saline administered into the abdominal cavity. To improve survival, animals were treated with the antibiotic ertapenem (20 μg/g bodyweight, Invanz^®^, Merck Sharp & Dohme Corp., Kenilworth, NJ, USA) intraperitoneally every 12 h for 72 h after AP. Control mice received physiological saline.

### Monitoring

After AP or NSI survival and disease severity were monitored every 6 h (except the night hours) for 72 h and afterwards every day until the end of the experiment. Disease severity was scored on the basis of (1) general appearance, (2) breathing frequency, (3) spontaneous and (4) provoked behaviour. Scoring points from 0  =   healthy to 3  =   severe alteration were given for each item and then summed up [32]. If the mice reached a severity score that indicated a disease point of no return (10 points), these mice were euthanized by cervical dislocation under deep anaesthesia immediately. Additionally, body temperature and weight were determined at the same time points.

In this study, none of the 29 animals subjected to the NSI procedure, but 12 out of 174 septic animals were euthanized and 47 died due to sepsis before reaching the humane endpoint. Most of them did not receive ertapenem (n = 31) as they were used as controls. This was necessary to analyze if ertapemen treatment has an impact on immunosuppression following sepsis. After excluding an impact all animals in the following experiments were treated with ertapenem to reduce suffering.

### Non-specific immunization (NSI)

To investigate the immune response to a mixture of antigens, mice were immunized intraperitoneally with 4 μg tetanus toxoid (Tetanol^®^, Novartis Behring, Marburg, Germany), 100 μg keyhole limpet hemocyanin (KLH, Calbiochem, Merck Millipore, Darmstadt, Germany), 25 μg CpG (24-mer, TIB Molbiol, Berlin, Germany, sequence: 5´-TsCsg sTsCsg sTsTsT sTsgsT sCsgsT sTsTsT sgsTsC sgsTsT), 2×10^5^ CFU heat-inactivated *S*. *aureus* strain JSNZΔspa (J. D. Fraser and S. Wiles, University of Auckland, New Zealand, S. Holtfreter; University of Greifswald, Germany) in 150 μL alum (Imject^®^, Thermo Fisher Scientific, Waltham, MA, USA).

### Immunosuppression: Test immunization

To study the immunosuppression due to sepsis or NSI, the animals were primed by intraperitoneal immunization with 100 μg 2,4,6-trinitrophenol (TNP_14_)-Ovalbumin (OVA, Biosearch Technologies, Petaluma, CA, USA) and 50 μg OVA (Sigma-Aldrich, St. Louis, MO, USA) in 105 μL alum suspension (Imject^®^, Thermo Fisher Scientific, Waltham, MA, USA) at the indicated time points after AP or NSI.

### Proliferation assay

To measure the antigen-specific recall response of T cells, splenocytes were restimulated *ex vivo* 14 days after priming. Splenocytes were isolated as described before [33]. 2×10^5^ splenocytes per well were stimulated with 1:3 serial dilutions of OVA (maximal concentration 100 μg/mL) or concanavalin A (maximal concentration 6 μg/mL; both stimulants were from Sigma-Aldrich, St. Louis, MO, USA). They were incubated for five days in RPMI 1640 (w/o glutamine, PAA Laboratories, Pasching, Austria) supplemented with 10% FCS (Biochrom, Berlin, Germany), 2 mM L-glutamine, 2 mM penicillin/streptomycin (both PAA Laboratories, Pasching, Austria), 1 mM sodium pyruvate, 1% non-essential amino acids MEM, 1% D-(+)-glucose (all Sigma-Aldrich, St. Louis, MO, USA) and 0.05 mM β-mercaptoethanol (Gibco, Thermo Fisher Scientific, Waltham, MA, USA). Afterwards, cells were incubated with 0.5 μCi methyl-3H-thymidine (Perkin Elmer, Waltham, MA, USA) for another 17 h. Cells were harvested to a glass fiber filter (Printed Filtermat A, Wallac, Perkin Elmer, Waltham, MA, USA) and radiation was measured via a Storage Phosphor Screen (Amersham Biosciences, GE Healthcare Life Sciences, Chalfont St Giles, Great Britain). Results were analyzed using ImageQuant (GE Healthcare, Chalfont St Giles, Great Britain). The T-cell recall response (cpm) was quantified at each stimulant concentration, and the area under the titration curve (AUC) was calculated.

### ELISpot

An ELISpot assay was performed to quantify OVA-specific antibody-secreting cells in the bone marrow of immunized mice. 96-well filter plates (MultiScreenHTS IP 96°, Merck Millipore, Darmstadt, Germany) were prepared first with 25 μL per well of 30% ethanol and washed with PBS three times. Afterwards, wells were coated with 50 μg/mL OVA (Sigma-Aldrich, St. Louis, MO, USA) and incubated overnight at 4°C; control wells were incubated with PBS only. After washing with PBS, the plates were blocked with 200 μL of 2% FCS/PBS per well for 2 h at 37°C in an incubator (5% CO_2_). Bone marrow cells were isolated by rinsing femur and tibia with 10% FCS/PBS using a 27-gauge syringe. After lysis of erythrocytes with *A*. *bidest*., cells were resuspended in RPMI 1640 (w/o glutamine, PAA Laboratories, Pasching, Austria) complemented with 10% FCS (Biochrom, Berlin, Germany), 2 mM L-glutamine and 2 mM penicillin/streptomycin (both PAA Laboratories, Pasching, Austria). At least 1×10^6^ cells per well (serial 1:2 dilution) were incubated overnight in an incubator (37°C, 5% CO_2_).

Cells were lysed by washing six times with wash buffer (PBS complemented with 2% FCS; Biochrom, Berlin, Germany) and 0.05% Tween 20^™^ (Sigma-Aldrich, St. Louis, MO, USA)). IgG binding was visualized by incubation with 1 μg/mL anti-mouse IgG-biotin (Dianova, Hamburg, Germany) for 2 h at 37°C, washing with wash buffer and incubating with 5 μg/mL streptavidin-horse radish peroxidase (HRP, Dianova, Hamburg, Germany) for 45 min at RT in the dark. After washing with PBS, 3-amino-9-ethylcarbazol dimethyl formamide acetate solution and H_2_O_2_ were added as substrate for 5 min, and the reaction was stopped with water. The analysis was carried out on an Immunospot series 5 versa ELISPOT Analyzer (CTL Europe GmbH, Bonn, Germany) with the ImmunoCapture^™^ image acquisition and ImmunoSpot^®^ analysis software V5.

### ELISA

OVA-specific serum IgG was measured by ELISA and normalized to a standard serum (arbitrary units, AU). 96-well microtiter plates (Nunc MaxiSorp^™^, Affymetrix eBioscience, Santa Clara, CA, USA) were coated with 0.5 μg OVA per well (Sigma-Aldrich, St. Louis, MO, USA) in coating buffer (Candor Bioscience GmbH, Wangen, Germany) overnight at 4°C, washed with PBS/0.05% Tween20^™^ and blocked with PBS/0.05% Tween20^™^/10% FCS. IgG binding was detected using goat anti-mouse IgG coupled to HRP (Southern Biotech, Birmingham, AL, USA) and BD OptEIATM TMB Substrate Reagent Set (BD, Franklin Lakes, NJ, USA). Optical density at 450 nm was measured with the Tecan Sunrise photometer (Tecan Group Ltd., Maennedorf, Switzerland).

The relative affinity of TNP-specific IgG antibodies was determined based on the quantification of binding to differently haptenized bovine serum albumin (BSA)–TNP_2_-BSA and TNP_26_-BSA (adapted from [34])–as the carrier protein. IgG binding to TNP-BSA (Biosearch Technologies, Petaluma, CA, USA) was measured by ELISA as described above, and relative affinity was calculated as the ratio of the binding to TNP_2_ to TNP_26_.

### Statistics

Group samples were analyzed for normal distribution. When data was normally distributed (parametric data) group differences were tested for significance with the Mann-Whitney and Dunn’s multiple comparison for selected pairs. In case of non-parametric data group differences were tested for significance with the Kruskal-Wallis and Dunn’s multiple comparison for selected pairs. Which test was done is indicated in each figure legend respectively. P values < 0.05 were assumed to be significant.

## Results

### The primary antigen-specific immune response was reduced after sepsis

**Fig 1:**
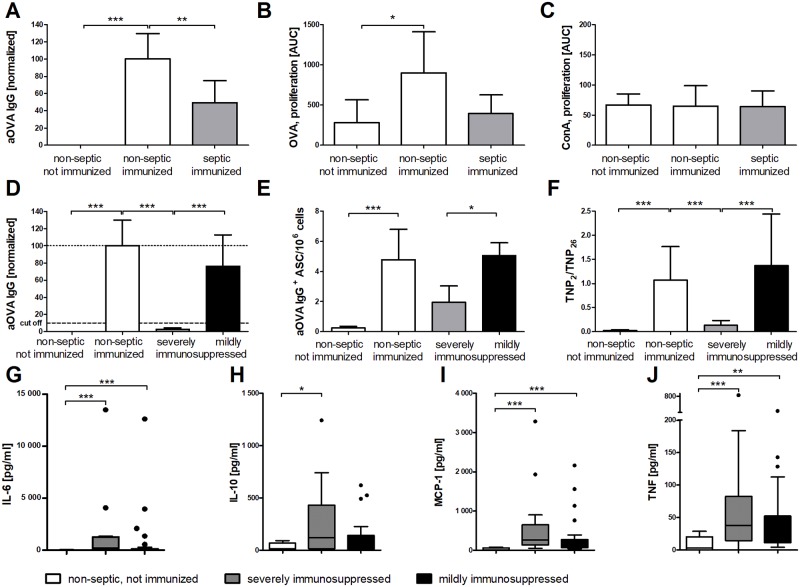
Suppression of the adaptive immune response after sepsis induction. Sepsis was induced in C57BL/6 mice, and on day 7, the animals were immunized intraperitoneally with TNP-OVA/OVA in alum (n = 47). Non-septic, not immunized animals (n = 32) and non-septic immunized animals (n = 25) served as controls. The antigen-specific immune response was determined 14 days after the immunization by measuring the serum concentration of anti-OVA IgG by ELISA. (A) The relative concentrations of OVA-specific IgG antibodies in serum normalized to a standard serum are shown (arbitrary units, AU). (B) The proliferative response to restimulation with OVA was measured by thymidine incorporation *ex vivo*. (C) The proliferative response to the mitogen concanavalin A (ConA) served as control. (D) Animals that had been immunized post-sepsis were divided into two groups: severely immunosuppressed mice (n = 17) had OVA-specific IgG concentrations below 10% of control values, while mildly immunosuppressed animals (n = 30) had serum concentrations greater than or equal to 10%. (E) Numbers of OVA-specific IgG-secreting cells in the bone marrow were determined in an ELISpot assay. (F) The relative antibody affinity of TNP-specific IgG was measured as binding to slightly haptenized TNP-BSA in comparison to highly haptenized TNP-BSA in an ELISA (adapted from [34]). (G-J) Blood was drawn 24 h after sepsis induction and cytokine concentrations of IL-6 (G), IL-10 (H), MCP-1 (I), and TNF (J) were quantified in the sera. Severely (n = 15) and mildly immunosuppressed (n = 29) animals are compared with non-septic, not immunized controls (n = 12). In the panels A-F means and 95% confidence intervals are shown. In the panels G-J box plots depict medians and quartiles. Group differences were tested for significance with the Kruskal-Wallis test and Dunn’s multiple comparison for selected pairs. Results were pooled from five separate experiments. *** P <0.001; ** P <0.01; * P <0.05.

Acute peritonitis (AP) in C57BL/6 mice was the sepsis model in this study [31]. Seven days after this “first hit”, animals were primed with TNP-ovalbumin and alum i.p., which served as test immunization or “second hit” for assessment of sepsis-induced immunosuppression. At this time point, the animals no longer showed signs of disease, e.g., hypothermia, stress or ruffled fur (S1A and S2B Figs). Fourteen days after the test immunization, non-septic control animals responded with the expected production of OVA-specific serum IgG. This response was significantly reduced in post-septic animals (Fig 1A). While antibiotic treatment with ertapenem improved sepsis survival by up to 30%, it did not restore the antigen-specific antibody response (S1C Fig). Following *ex vivo* restimulation of splenocytes with OVA, the antigen-specific proliferation was reduced in post-septic vs. non-septic control mice (Fig 1B). In contrast, the response to the mitogenic stimulus concanavalin A (ConA) was not altered (Fig 1C).

To identify parameters correlated with the degree of immunosuppression, we divided septic animals into two groups according to the amount of OVA-specific IgG produced after immunization; animals producing less than 10% anti-OVA IgG compared to the mean in immunized control animals were defined as severely immunosuppressed the remainder as mildly immunosuppressed. The range of the specific IgG responses to OVA in severely immunosuppressed animals did not overlap with those in non-septic immunized mice (Fig 1D). Severe immunosuppression was associated with lower numbers of OVA-specific IgG-secreting cells (ASC) in the bone marrow (Fig 1E), and severely immunosuppressed mice showed significantly less affinity maturation of the TNP-specific IgG response compared to mildly immunosuppressed and non-septic control mice (Fig 1F). The relative affinity of the specific serum IgG antibodies was quantified by comparing the IgG binding to low-haptenized TNP-BSA with that to high-haptenized TNP-BSA (adapted from [34]).

Remarkably, 24 h after AP, severely immunosuppressed animals tended to have higher concentrations of IL-6, IL-10, MCP-1 and TNF than did mildly immunosuppressed mice (Fig 1G–1J), while IFN-γ and IL-12p70 remained low in all animals. We conclude that the degree of immunosuppression is positively correlated with inflammation at the early stage of sepsis.

### The role of Tregs in sepsis-induced immunosuppression

**Fig 2:**
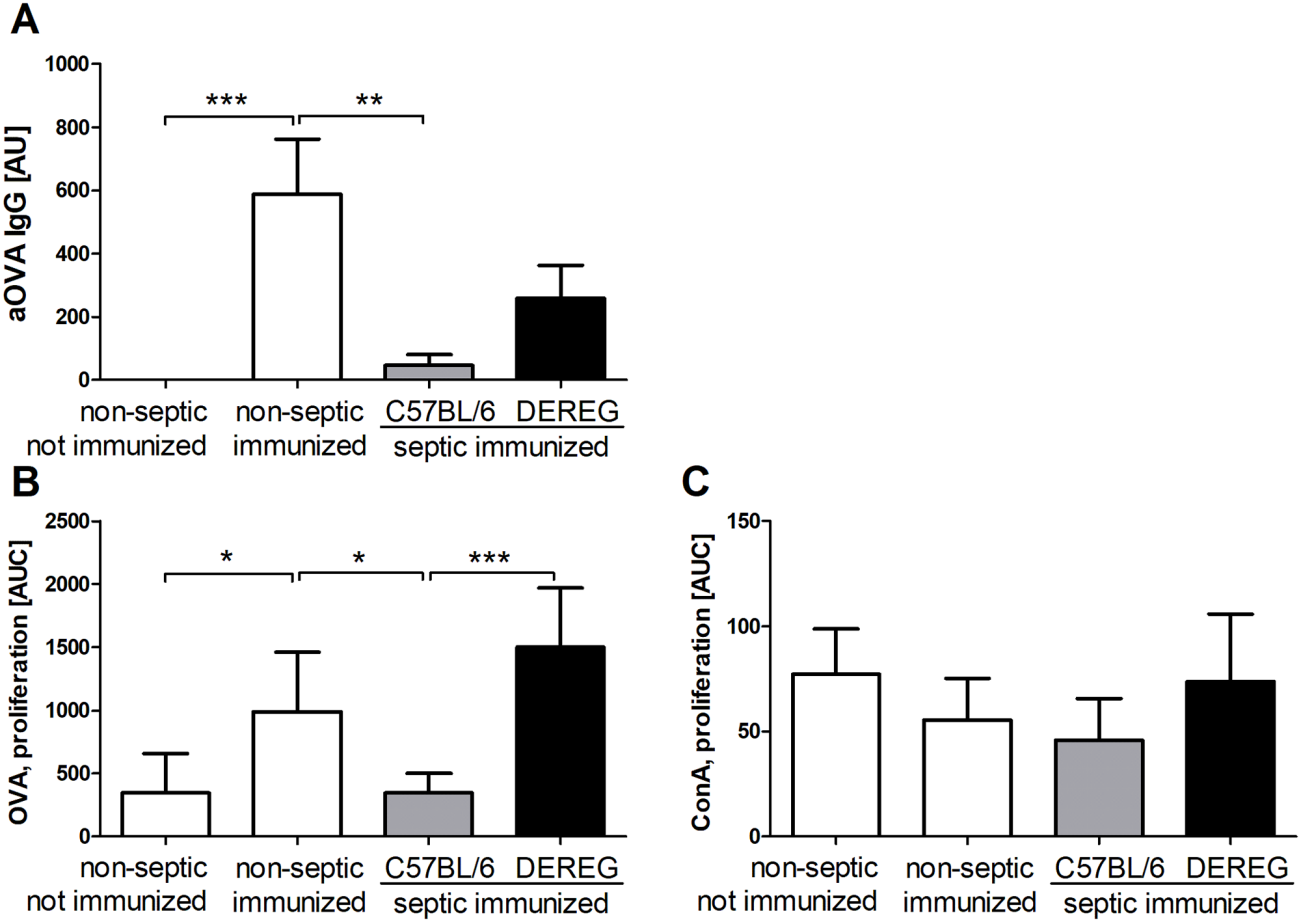
Treg depletion prior to immunization of post-septic animals. In DEREG mice (n = 15) and C57BL/6 wild-type controls (n = 9) sepsis was induced, and on days 5 and 6 the animals were given 1 μg of diphtheria toxin intraperitoneally to deplete Tregs. On day 7, the animals were immunized intraperitoneally with TNP-OVA/OVA in alum. Non-septic, not immunized animals (n = 11) and non-septic immunized animals (n = 10) served as controls. (A) 14 days after immunization, the antigen-specific immune response was determined by the concentration of anti-OVA IgG in the serum via ELISA and normalized to a standard serum (arbitrary units, AU). (B) The proliferative response of splenocytes to restimulation with OVA was measured by thymidine incorporation *ex vivo*. (C) The proliferative response to the mitogen concanavalin A (ConA) served as control. Means and 95% confidence intervals are shown. Group differences were tested for significance with the Kruskal-Wallis test and Dunn’s multiple comparison for selected pairs. Pooled results from three separate experiments with the same tendency are shown. *** P <0.001; ** P <0.01; * P <0.05.

To investigate the role of Foxp3^+^ Tregs in sepsis-induced immunosuppression, we used DEREG mice (**de**pletion of **reg**ulatory T cells), which express a fusion protein of eGFP and the human diphtheria toxin (DT) receptor under the control of the *foxp3* promoter [35]. This makes it possible to identify CD4^+^Foxp3^+^ Tregs by means of green fluorescence and selectively deplete them *in vivo* by administration of DT (S2 Fig). T_fr_ cells also express Foxp3 and are, hence, susceptible to DT-mediated depletion in DEREG mice [36]. In DEREG mice, the reaction to AP, including survival rate, symptoms (disease severity score), bacterial load and cytokine response, were unaltered compared to wildtype (WT) animals (S3 Fig). Previous experiments demonstrated an important role of Tregs in limiting the early hyperinflammatory phase of sepsis [27]. To preserve this Treg-mediated counter regulation of systemic inflammation, the cells were depleted after resolution of sepsis symptoms, directly before the test immunization (on day 5 and 6 after sepsis induction). Treg depletion tended to restore the OVA-specific IgG response in septic animals (Fig 2A). The proportion of severely immunosuppressed animals was lower in Treg depleted (3 out of 15) than in Treg competent mice (5 out of 8). The antigen-specific proliferative response of splenocytes was fully restored in septic mice by Treg depletion (Fig 2B). Splenocyte proliferation in response to the mitogen ConA was not affected by sepsis and it did not change significantly following Treg depletion (Fig 2C).

### Despite antigen-specific immunosuppression, a strong humoral immune response takes place during sepsis

**Fig 3:**
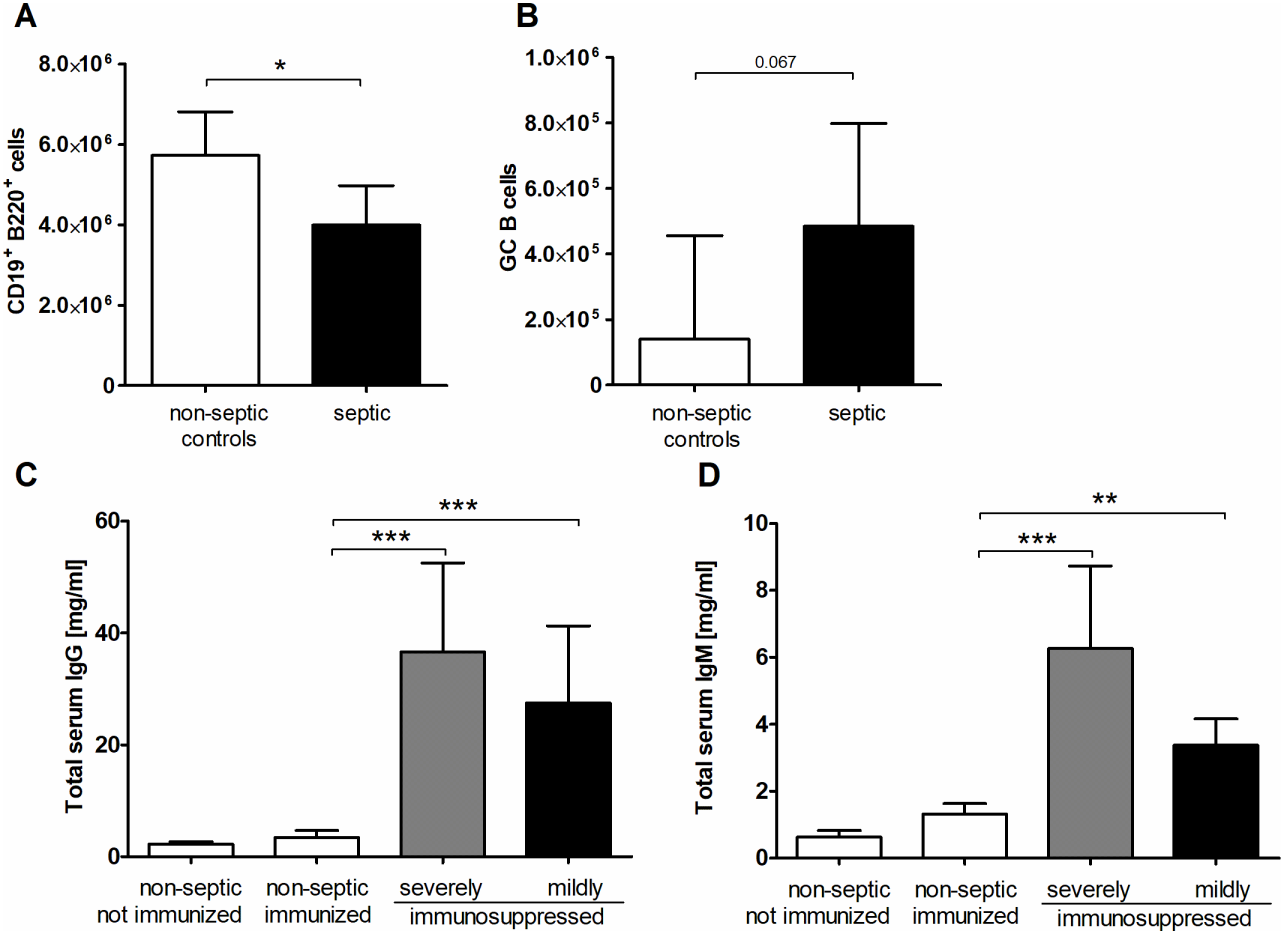
Sepsis elicits a strong serum antibody response. Sepsis was induced in C57BL/6 mice by AP (n = 7) and non-septic mice served as control (n = 3). After seven days, spleens were harvested and numbers of CD19^+^ B220^+^ total B cells (A) and B220^+^ GL7^+^ IgD^-^ CD95^hi^ CD73^int^ germinal center (GC) B cells (B) were determined by flow cytometry. Gating strategies can be found as supporting information (S4 Fig). Seven days after AP mice were immunized intraperitoneally with TNP-OVA/OVA in alum, and the animals were divided into severely immunosuppressed animals (n = 15) and mildly immunosuppressed (n = 29) as depicted in Fig 1D. Non-septic, not immunized animals (n = 23) and non-septic immunized mice (n = 19) served as controls. 14 days after the test immunization, concentrations of total IgG (C) and IgM (D) in serum were measured by Luminex technology. Means and the 95% confidence interval are shown. Group differences were tested for significance with the Mann-Whitney (A, B) or the Kruskal-Wallis test and Dunn’s multiple comparison for selected pairs (C, D). Results from four separate experiments with the same tendency were combined. *** P <0.001; ** P <0.01; * P <0.05.

During sepsis, B cells are depleted by apoptosis [8], as was also observed in the spleen in this study (Fig 3A). Nevertheless, AP induced the generation of splenic germinal center B cells (Fig 3B). The gating strategies for total B cells (CD19+ B220+) and germinal center B cells (B220^+^ GL7^+^ IgD^-^ CD95^hi^ CD73^int^[37–40]) are shown in the supporting material (S4 Fig). Moreover, in stark contrast to their inability to mount a primary antigen-specific IgG response 7 days after AP, septic animals had strongly increased concentrations of total serum IgG and IgM. Interestingly, the increase of serum IgG and IgM appeared to be inversely related to the degree of antigen-specific immunosuppression (Fig 3C and 3D).

### Immunization with a complex antigen mixture leads to adaptive immunosuppression in the absence of sepsis

**Fig 4:**
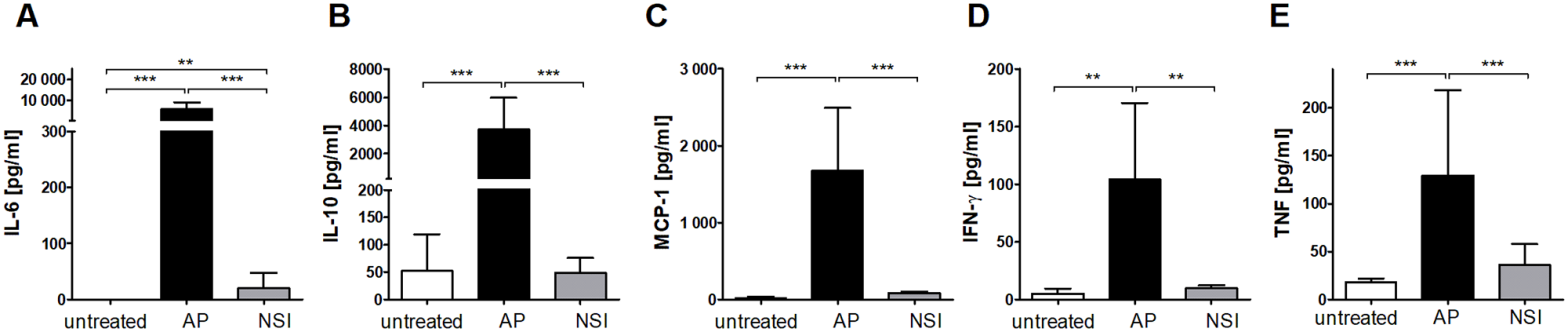
Serum cytokine concentrations 24 h after NSI and AP. C57BL/6 mice were non-specifically immunized (n = 29), or sepsis was induced by AP (n = 23). Untreated animals (n = 12) served as controls. 24 h after NSI or AP, respectively, cytokine concentrations of IL-6 (A), IL-10 (B), MCP-1 (C), IFN-γ (D) and TNF (E) and were determined in serum by CBA. Means and the 95% confidence intervals are shown. Group differences were tested for significance with the Kruskal-Wallis test and Dunn’s multiple comparison for selected pairs. Pooled results from two separate experiments are shown. *** P <0.001; ** P <0.01.

We reasoned that the strong humoral immune response induced by sepsis may interfere with subsequent priming of an antigen-specific response by competing for limited niches in the lymphoid follicles. To experimentally separate the impact of a strong antigenic stimulus from the severe form of the disease caused by the multi-factorial septic insult in AP, we adopted the procedure of non-specific immunization (NSI) described by Xiang *et al*. [29] and immunized mice with a mixture of Tetanol^®^, keyhole limpet hemocyanin (KLH), heat-inactivated *S*. *aureus* with CpG and alum as adjuvants. The procedure is termed “non-specific” because the complex antigen mixture does not contain the test antigen TNP-OVA. As expected, all animals survived and they did not develop disease symptoms after NSI (S5 Fig). In stark contrast to sepsis, NSI-treated mice showed only minimal increases in serum cytokine concentrations and, hence, did not mount a systemic inflammatory response (Fig 4).

**Fig 5:**
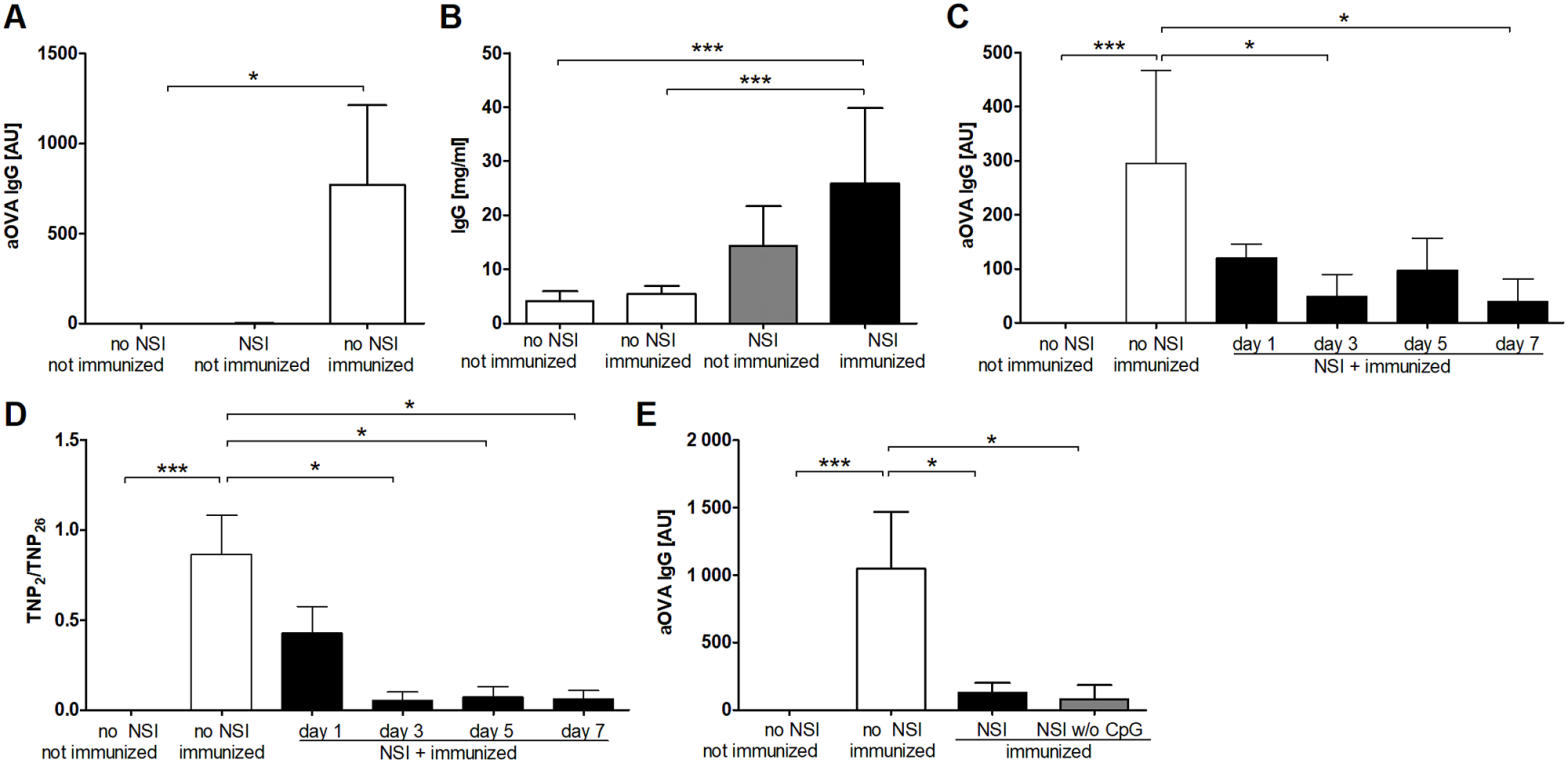
Suppression of the adaptive immune response after non-specific immunization. (A, B) NSI alone did not induce OVA-specific serum antibodies but increased total serum IgG. OVA-specific IgG (A) and total serum IgG (B) were measured 21 days after NSI (n = 3). Animals without NSI and immunization (n = 3 and 6), only immunized animals (n = 5 and 11), and animals immunized 7 days after NSI (n = 6 and 11) served as controls. (C, D) NSI reduced the specific response to antigen priming. C57BL/6 mice were non-specifically immunized (NSI, adapted from [29]). At the indicated time points after NSI, the animals were primed intraperitoneally with TNP-OVA/OVA in alum (n = 5–6). Animals without NSI and immunization (n = 6) and animals immunized without prior NSI (n = 5) served as controls. The antigen-specific immune response was measured as the concentration of anti-OVA IgG in the serum 14 days after priming with OVA by an ELISA. (C) The relative concentrations of OVA-specific IgG antibodies in serum normalized to a standard serum (arbitrary unit, AU) are shown. (D) The relative antibody affinity of TNP-specific IgG was measured as binding to slightly haptenized TNP-BSA in comparison to highly haptenized TNP-BSA in an ELISA (adapted from [34]). (E) The NSI-induced immune suppression was independent of CpG. C57BL/6 mice were treated with NSI containing CpG (NSI, n = 12) or not (NSI w/o CpG, n = 6). Seven days later, they were immunized intraperitoneally with TNP-OVA/OVA in alum (OVA). Mice without NSI and immunization (n = 7) and animals immunized only (n = 9) served as controls. The antigen-specific immune response was determined as described for panel C. In the panels A-D means and 95% confidence intervals are shown. Kruskal-Wallis test and Dunn’s multiple comparison for selected pairs. In panels B and E pooled results from two separate experiments are shown. *** P <0.001; * P <0.05.

NSI did not induce OVA-specific antibodies (Fig 5A). Nevertheless, NSI-treated animals generated a strong IgG response similar to that observed in sepsis (Fig 5B). Remarkably, even in the absence of disease symptoms, NSI strongly interfered with the antibody response to priming with TNP-OVA. Both the concentrations of specific IgG and the affinity for the antigen were affected to a similar degree as in sepsis (Fig 5C and 5D). While on day 1 after NSI all five tested animals were mildly suppressed, 4 animals were severely and 11 mildly immunosuppressed between days 3 and 7 following NSI. It has previously been described that systemic administration of CpG can suppress a subsequent adaptive immune response [41]. However, when applied without CpG, the inhibitory effects of NSI on the response to priming were fully preserved in terms of antigen-specific antibodies as well as numbers of ASC in the bone marrow (Fig 5E).

### Role of Tregs in immunosuppression after NSI

**Fig 6:**
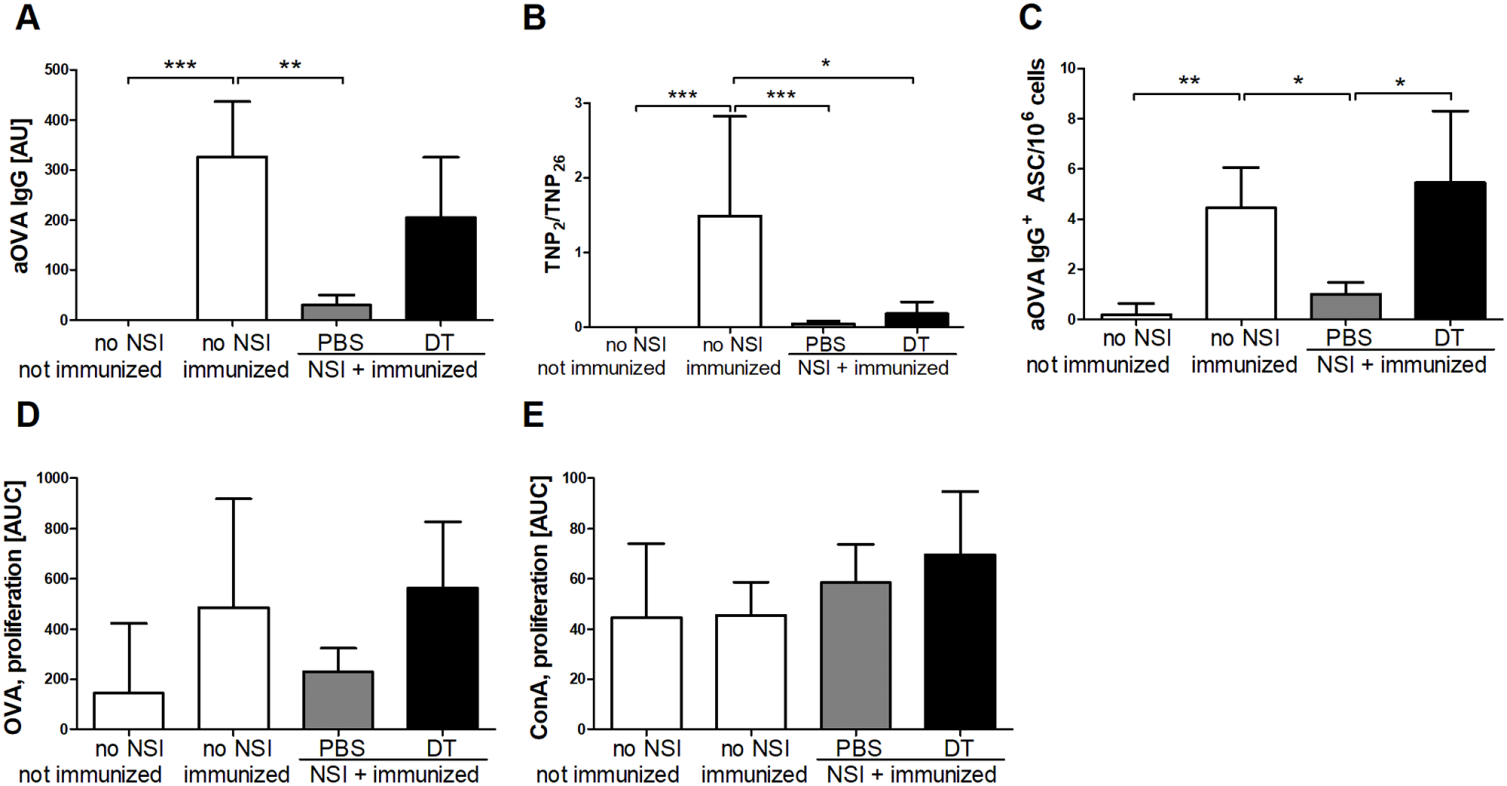
Treg depletion prior to immunization of NSI-treated mice. DEREG mice (n = 12) were non-specifically immunized (NSI, adapted from [29]). On days 5 and 6, the animals were given 1 μg of diphtheria toxin intraperitoneally to deplete Tregs; control mice received the same volume of PBS (n = 12). Seven days after NSI, the animals were primed by intra-peritoneal immunization with TNP-OVA/OVA in alum. Animals without NSI and immunization (n = 6) and animals immunized with TNP-OVA/OVA without prior NSI (n = 9) served as controls. The relative concentration of anti-OVA IgG (A) and the relative antibody affinity (B) were measured as described in the caption of Fig 5. (C) Numbers of OVA-specific IgG-secreting cells in the bone marrow were determined in an ELISpot assay. (D) The proliferative response of splenocytes to restimulation with OVA was measured in a proliferation assay *ex vivo*. (E) The proliferative response to concanavalin A (ConA) served as control. Means and the 95% confidence interval are shown. Group differences were tested for significance with the Kruskal-Wallis test and Dunn’s multiple comparison for selected pairs. Results from two separate experiments were combined. *** P <0.001; ** P <0.01; * P <0.05.

On day 7, the time point of the test immunization, the fraction of Foxp3^+^ Tregs among CD4^+^ T cells was similar in NSI and in post-septic mice (13.52 ± 2.19% and 13.99 ± 1.96%, respectively). To elucidate the role of Tregs in NSI-mediated immune suppression, the DEREG model was once again applied to deplete Foxp3^+^ Tregs following the same time course as in the sepsis experiments. The depletion of Tregs tended to increase the production of OVA-specific IgG, but this effect was not significant (Fig 6A). A similar tendency was observed in terms of the affinity maturation of IgG antibodies binding to TNP (Fig 6B). Only the numbers of OVA-specific IgG^+^ ASCs in the bone marrow were completely restored after depletion of Tregs (Fig 6C). Finally, the response of splenocytes to restimulation with OVA showed the same tendency, but the effects were not significant (Fig 6D). The proliferative response to the mitogenic stimulus ConA was not affected by NSI or Treg depletion (Fig 6E). Together, these data suggest that Tregs also contribute to immunosuppression after NSI.

## Discussion

We studied inhibition of the adaptive immune response during sepsis by using primary immunization with antigen and adjuvant as a second hit and observed reduction of the antigen-specific humoral (antibody and ASC) and cellular responses (T cells). Remarkably, NSI induced a similar suppression of the adaptive immune response, showing that it can occur independently of surgical trauma and systemic bacterial dissemination, which in sepsis cause life-threatening illness.

Our finding of sepsis-mediated suppression of the response to antigen priming is in agreement with observations in CLP reported by Mohr *et al*., showing that adaptive immune suppression is a robust phenomenon occurring in different sepsis models [20]. The inability to mount a primary immune response is probably an important factor in the failure to control secondary infections, which has been identified as a major cause of death in septic patients and experimental animals [42–44].

Regarding the mechanisms of sepsis-induced immune suppression, there is evidence of passive immunodeficiency as well as active suppression [45–51]. Cell death is widespread in sepsis, and its inhibition improves survival in experimental settings [52,53]. However, adoptive transfer of naive DCs, B cells or CD4^+^ T cells did not reverse sepsis-mediated immunosuppression in the study by Mohr et al. [20]. Therefore, death of these cell populations was not decisive in CLP.

In contrast, depletion of Foxp3^+^ Tregs partially restored adaptive immune competence in AP. It is well documented that the proportion of Foxp3^+^ Tregs increases during sepsis due to their relative resistance to apoptosis [27,54–56] or potential transdifferentiation from Th17 cells into Treg during bacterial infections [57]. Moreover, the cells become strongly activated in human as well as in experimental sepsis [58], and they increase their suppressive potential [27,59]. This was also the case in AP, where Tregs were rapidly activated to express the surface markers CD69, CD25 and CTLA-4 [58]. Tregs have a dual role during infection. On the one hand, they mitigate inflammation, which in sepsis is essential for the return to homeostasis [27], and on the other, they counteract mechanisms of bacterial elimination. In sepsis, this promotes dangerous sequelae, namely chronification of the primary infection as well as the development of secondary infections [60]. Hence, Tregs may be a double-edged sword in infection [15,18,56,59–66]. In this study, care was taken to preserve the initial anti-inflammatory function of the Tregs, which were depleted in septic DEREG mice only after the animals had fully recovered from the symptoms of sepsis, immediately before the priming immunization. Under these conditions, the generation of antigen-specific IgG tended to increase again, and the antigen-specific proliferation of T cells was fully restored. This underlines the importance of appropriately timing immune interventions in sepsis.

Despite the loss of approximately 30% of CD19^+^B220^+^ B cells in the spleen during AP, there was a sharp increase in total IgM and IgG serum levels. Massive apoptosis of B cells [8,43,67,68], as well as large increases in serum Ig concentrations [20,28], have been reported by numerous groups, both in septic humans and experimental animals. This is in line with increases in the proportions and absolute numbers of both germinal center B cells and splenic plasma cells observed in AP (the present study) as well as in CLP [20,69]. As the increase in total serum IgG tended to be more pronounced in the most severely immunosuppressed animals, we reasoned that competition with B cells responding to the sepsis-causing agents might contribute to the failure of OVA-specific B cells to mount a primary immune response during or after sepsis, since the niches for germinal center reactions are limited [24].

To test this hypothesis, the poly-antigenic challenge typical of sepsis was imitated by "non-specific" immunization (NSI, adapted from [29]). Remarkably, this mild intervention reproduced both the strong humoral immune response characterizing sepsis and the failure to specifically respond to a subsequent priming immunization with TNP-OVA in alum. However, as expected and in stark contrast to sepsis, NSI neither induced symptoms of illness nor systemic increases of inflammatory cytokines such as IFN-γ, MCP-1 or TNF. Only the concentration of IL-6 was slightly increased after NSI. This clearly demonstrates that adaptive immunosuppression can be separated from the severe form of the illness, bacterial dissemination, and surgical trauma, which are associated with sepsis but not with NSI. The independence of post-septic immunosuppression from trauma was also described by Murphey *et al*. during CLP [70]. In contrast, various research groups showed a correlation between surgical trauma and a subsequent immunosuppression in humans [71–73] and mice [74]. The results of the current study make a case for antigen competition as a contributing factor in sepsis-induced immunosuppression.

Similar to the situation in sepsis, Tregs moderately contributed to immunosuppression following NSI. Depletion of these cells immediately before the test immunization tended to increase the OVA-specific IgG response (35%) as well as the antibody affinity and it restored the number of specific antibody secreting cells to control values. It is known that Tregs can suppress the B cell response directly via CTLA-4 and TGF-β [75,76]. T_fr_ cells, expressing typical markers of follicular T cells (Bcl6, CXCR5) as well as Tregs (Foxp3, CD25, GITR, CTLA-4), are of particular interest in this context. They limit the germinal center response, including affinity maturation in the secondary lymphoid organs [77,78]. Since our intervention targeted Foxp3, T_fr_ cells, which express this transcription factor, were probably also deleted [36].

The role of Tregs and T_fr_ in humoral immunity is controversially discussed, because depletion experiments yielded conflicting results, either enhancement or inhibition of responses to foreign antigens [24]. Tregs control both self- and foreign-reactive follicular helper T cells (T_fh_) and B cells and the duration of Treg-depletion appears to have a strong influence on the outcome: Wing and co-workers studied T_fh_ cells which are required for specific antibody responses. They reported an increased antigen-specific vaccine response upon transient Foxp3^+^ Treg/T_fr_ depletion during priming. Conversely, depletion of long duration reduced vaccine antigen-specific T_fh_ cell numbers, while non-specific T_fh_ cells expanded strongly. The authors propose that long-term depletion of Foxp3^+^ Tregs unleashes autoreactive T cells, which become activated and compete with the antigen-specific cells [36]. In the present study, the depletion of Foxp3^+^ Tregs was transient, and, in agreement with the observations of the group of Wing, this facilitated the vaccine response.

If Tregs exert a suppressive effect on adaptive immunity in sepsis and NSI, this may open avenues for therapeutic intervention, for instance, as currently being pioneered in tumor therapy [79–81]. In Foxp3^+^ Tregs this transcription factor is essential for their development and function [82–85]. Down-regulation of Foxp3 expression by small interfering RNA (siRNA) restored the proliferation of CD4^+^ T cells after CLP [55]. Another possibility is interference with Treg functions. Tregs suppress via the secretion of anti-inflammatory cytokines (e. g. IL-10), the competition for growth factors (IL-2), cytolysis or cell contact involving inhibitory receptors (e. g. CTLA-4, PD-1, GITR) [86–88]. Nascimento *et al*. blocked GITR, thus increasing the proliferative response of CD4^+^ T cells after CLP, and significantly reducing mortality after secondary infection with *Legionella pneumophila* [15]. Similarly, interference with CTLA-4 function improved survival in bacterial and fungal sepsis [61,63] as well as in pneumococcal pneumonia [65]. Inoue *et al*., on the other hand, reported that a high dose of anti-CTLA-4 antibody reduced survival in septic mice [61]. It should be noted that CTLA-4 is not only expressed by Tregs but also by activated effector T cells [86,89,90].

In sepsis, PD-1 expression is also increased on the surface of different T cell subpopulations, including Tregs [62,63,91,92]. The therapeutic blockade after CLP improved survival, reduced lymphopenia and preserved delayed type hypersensitivity reactions, a marker of immune competence [62]. Moreover, inhibition of heme oxygenase (HO)-1 after CLP lead to better survival and bacterial clearance after secondary *Pseudomonas aeruginosa* infection in mice. HO-1 has a regulatory effect on differentiation and function of T cells and anti-inflammatory properties. Its inhibition after CLP resulted in decreased Treg population and attenuated expression of inhibitory co-stimulatory molecules [93]. Finally, neutralization of IL-10, a key factor in Treg-mediated suppression [94,95], improved survival in a mouse model [18,64] and increased the secretion of pro-inflammatory cytokines by Th1 cells [66].

In conclusion, our data suggest that, besides cell death, active suppressive mechanisms, such as cellular competition and Treg activation, should be considered in sepsis-related immunodeficiency. Moreover, the failure of immune priming following NSI clearly shows that many aspects of "sepsis-induced" immunosuppression can be separated from disease symptoms, surgical trauma, and systemic bacterial dissemination.

## Supporting information

S1 Fig Disease severity, body temperature and ertapenem treatment during AP. Sepsis was induced in C57BL/6 mice by the AP procedure, and mice were treated with ertapenem intraperitoneally every 12 h (n = 28). Non-septic animals (n = 3) served as controls. (A) Over a period of 72 h sepsis severity was determined as the sum of scores assessing general appearance, breathing, provoked and spontaneous behavior. (B) At the same time, body temperature was measured. (C) In separate experiments sepsis was induced in C57BL/6 mice which received ertapenem as antibiotic treatment (n = 20) or were treated with the same volume of physiological saline as control (n = 27). Seven days after AP, the animals were immunized intraperitoneally with TNP-OVA/OVA in alum. Non-septic, not immunized mice (n = 32) and non-septic immunized animals (n = 25) served as controls. The antigen-specific immune response was determined by ELISA 14 days after immunization by measuring the relative serum concentration of anti-OVA IgG normalized to a standard serum (arbitrary unit, AU). In panels A and B means and standard errors are shown for combined results of two independent experiments. In panel C pooled results of five experiments are depicted and the means are indicated.
(TIF)

S2 Fig Verification of Treg depletion in the blood seven days after AP and composition of the spleen seven days after NSI and AP. Sepsis was induced in DEREG mice (n = 4) and C57BL/6 wild-type controls (n = 4) by AP, and on days 5 and 6, the animals were given 1 μg of diphtheria toxin intraperitoneally to deplete Tregs. Non-septic mice served as controls (n = 5). Depletion of Treg was confirmed in blood by flow cytometry on day 7 after AP. (A) The proportion of Foxp3^+^ Tregs in the CD4^+^ T-cell population is shown as median and quartiles. Group differences were tested with the Kruskal-Wallis test and Dunn’s multiple comparison for significance. ** P <0.01. (B) Representative density plots showing the Foxp3^+^ CD4^+^ Treg population before and after depletion are shown.
(TIF)

S3 Fig Survival, stress levels, bacterial load, and cytokine response at the beginning of sepsis were similar in WT and DEREG mice. In DEREG mice (n = 53) and C57BL/6 wild-type controls (n = 36), sepsis was induced by 18G AP. (A) The survival of the animals was closely monitored for three days. (B) In addition, sepsis severity was determined as a sum of scores assessing general appearance, breath, provoked and spontaneous behavior. (C) At the same time, the body temperature was measured. (D-H) 24 h after AP, cytokine concentrations of IL-6 (D), IL-10 (E), MCP-1 (F), IFN-γ (G), and TNF (H) were determined in serum by CBA. (I) Moreover, mice were analyzed for systemic dissemination of bacteria by measuring the bacterial load in the blood 24 h after AP. In panels A-C means and standard errors are shown and results represent four independent experiments. In panels D-I the median is shown. Group differences were tested for significance using the Mann-Whitney test.
(TIF)

S4 Fig Gating for B cells and germinal center B cells. Sepsis was induced in C57BL/6 mice by AP. After seven days, B cells and germinal center (GC) B cells in the spleen were determined by flow cytometry. The gating strategies for CD19^+^ B220^+^ B cells (A) and B220^+^ GL7^+^ IgD^-^ CD95^hi^ CD73^int^ GC B cells (B) are shown.
(TIF)

S5 Fig NSI did neither cause disease symptoms nor death. C57BL/6 mice were non-specifically immunized (NSI, n = 29). Untreated animals (n = 12) and animals in whom sepsis has been induced by AP (n = 23) served as controls. (A) The survival of the animals was closely monitored over three days. Survival differences were analyzed with log rank test. (B) In addition, the severity of illness was determined as a sum of scores assessing general appearance, breath, provoked and spontaneous behavior. (C) At the same time the body temperature of the animals was determined. Means and standard errors are shown. The results are pooled from two independent experiments. Group differences between "untreated" and "NSI" (#) or "AP" and "NSI" (*) were tested for significance with the Mann-Whitney test. * / # P <0.05; ** / ## P <0.01; *** / ### P <0.001.
(TIF)
