# Supplementary figures and images for: Polymicrobial sepsis and non-specific immunization induce adaptive immunosuppression to a similar degree
Source: PLoS One. 2018 Feb 7;13(2):e0192197. doi: 10.1371/journal.pone.0192197 (PMC5802895; doi:10.1371/journal.pone.0192197)

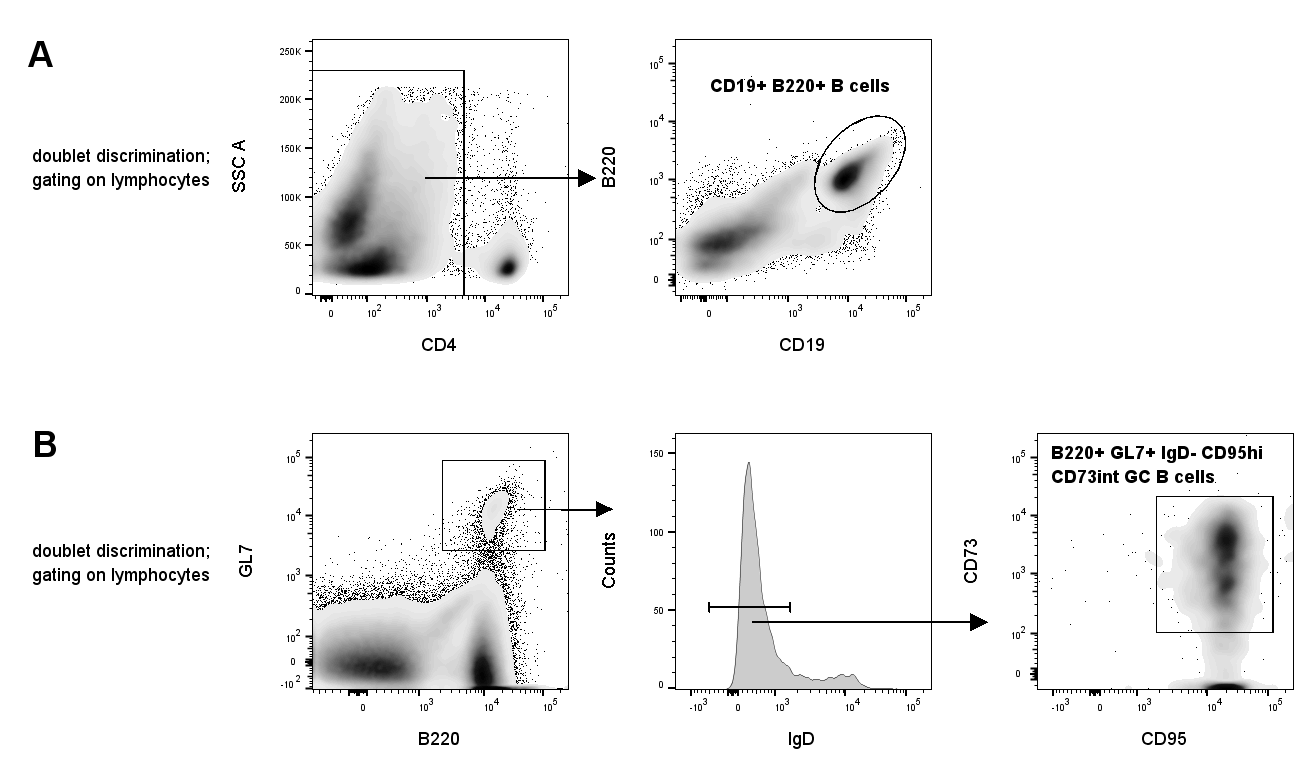

Supplement: S4 Fig — Sepsis was induced in C57BL/6 mice by AP. After seven days, B cells and germinal center (GC) B cells in the spleen were determined by flow cytometry. The gating strategies for CD19+ B220+ B cells (A) and B220+ GL7+ IgD- CD95hi CD73int GC B cells (B) are shown. (TIF) [file pone.0192197.s004.tif]
